# Impact of low and high lipopolysaccharide doses on the early expression of immune related genes and transcription factors in chicken macrophage-like HD11 cells

**DOI:** 10.1016/j.psj.2025.106229

**Published:** 2025-12-09

**Authors:** Samuel C.G. Jansseune, Jürgen van Baal, Fany Blanc, Aart Lammers

**Affiliations:** a Animal Nutrition Group, Department of Animal Sciences, Wageningen University & Research, Wageningen, the Netherlands; b Adaptation and Physiology Group, Department of Animal Sciences, Wageningen University & Research, Wageningen, the Netherlands; c Université Paris‐Saclay, INRAE, AgroParisTech, GABI, Jouy‐en‐Josas, France; d Idena, Sautron, France

**Keywords:** HD11 cells, Early transcriptome, Transcription factor, Pathways

## Abstract

This study aimed to investigate how early transcriptional reprogramming of the chicken macrophage-like HD11 cell line differs when exposed to a low and high dose of lipopolysaccharide (**LPS**). Transcriptome analysis was performed on cells stimulated with 0, 3 and 300 ng/ml *E. coli* LPS for 5 h. After the processing steps, 10796 genes remained. Compared to the negative control, 11.8 and 16.3 % were upregulated at 3 and 300 ng and 25.8 and 44.1 % of the genes were downregulated. Subsequently, gene set enrichment analysis of Kyoto Encyclopedia of Genes and Genomes pathways demonstrated that, compared to the negative control, LPS at 3 and 300 ng/ml upregulated and downregulated similar pathways, out of which only three were significantly more affected at 300 than at 3 ng/ml LPS. These three enriched pathways were the toll-like receptor, cytokine-cytokine receptor interaction and C-type lectin receptor signalling pathways. Five transcription factors related to immune genes (PITX2, TCF7L2, NFIL3, NFATC1 and FOXO1) were downregulated at the high dose of LPS only and retrieved in the regulatory network between the differentially expressed immune related genes and transcription factors. Six other transcription factors were only retrieved in the 300 vs 3 ng/ml network: HMGB1, MEF2C, IKZF1, and PITXA, which were downregulated, and SRF and FOS, which were upregulated. These 11 transcription factors may account for the differences in the intensity of the cell response to low and high doses of LPS. Our results indicate that while the expression of transcription factors is largely similar, there are some differences between low and high doses of LPS. These differences could have implications when using LPS-stimulated HD11 cells as a model to study potential feed additives in poultry. Based on this knowledge we recommend considering the use of multiple LPS doses when using HD11 cells as an *in vitro* model to study the effects of dietary or pharmaceutical compounds on inflammation in chicken macrophages.

## Introduction

The innate immune system is the first line of defence against invading pathogens. Macrophages fulfil a crucial role in innate immunity, and have a wide range of functions (Ciraci et al., 2010; Sokolowska et al., 2015). For example, macrophages eliminate pathogens by phagocytosis, produce antimicrobial molecules, present antigens to T cells, secrete immune-modulating cytokines, but are also involved in tissue repair and wound healing (Klasing, 1998; Qureshi, 2003; Guimarães et al., 2011; Mantovani et al., 2013). In addition, it was recently shown that the concept of innate immune training also applies to chicken macrophages, giving them potential as a target for dietary immunomodulatory approaches (Netea et al., 2011; Verwoolde et al., 2021).

Macrophages are activated by pathogens or microbe-derived conserved molecular patterns (**MAMP**) and through the recognition of damage-associated molecular patterns (**DAMP**), derived from damaged host cells (Zhang and Mosser, 2008). One of the best characterised MAMP is the bacterial endotoxin lipopolysaccharide (**LPS**) (Zhang and Mosser, 2008). This component is located in the cell wall of Gram-negative bacteria and has extensively been used to study the inflammatory response of macrophages, both *in vivo* and *in vitro*. LPS is a well-known ligand for toll-like receptor (**TLR**) 4 (Brownlie and Allan, 2011). Upon stimulation, TLR-4 activates nuclear factor-κB and mitogen-activated protein kinases, eventually leading to the production of inflammatory mediators, such as nitric oxide (**NO**), prostaglandins and cytokines in macrophages (Crippen, 2006; Lin et al., 2017).

Immortalized macrophage-like cell lines are often used as a convenient model to pre-screen the effects of various types of compounds (e.g. pro-, syn- and postbiotics and phytochemicals) for use as feed additives (Paradowska et al., 2022). The dietary compounds are tested in combination with a single, usually high, dose of LPS to determine if the compounds could interfere with the LPS-evoked inflammatory response. However, in murine macrophages, it was shown that a low dose of LPS activates the MyD88-independent TRAM-dependent pathway, causing low-grade inflammation, while a high dose of LPS induced inflammation through the MyD88-dependent pathway (Naler et al., 2022). In contrast to mammalian macrophages, TLR4 stimulation of chicken macrophages does not activate the MyD88-independent pathway (Keestra and van Putten, 2008), because the chicken genome lacks TRAM (Brownlie and Allan, 2011). This may explain the lower sensitivity of chickens to LPS-induced sceptic shock when compared to many mammalian species as reported by Berczi et al. (1966). Therefore, chicken may have a specie specific response (Keestra and van Putten, 2008). The transcriptional reprogramming of chicken macrophages upon activation by different doses of LPS has not been published in the literature yet, whereas it is valuable for the screening of dietary compounds.

In the present study we investigated, therefore, how the stimulation of HD11 macrophage like cells with a low and high LPS dose results in differential transcriptional reprogramming. The results can be helpful for the design of *in vitro* experiments aiming to investigate the effects of dietary or pharmaceutical compounds on inflammation in chicken macrophages.

## Materials and methods

### Cell culture

Chicken macrophage-like cell line HD11 constitutes an established chicken myelomonocytic line transformed by the MC29 virus (Beug et al., 1979). HD11 cells, kindly provided by Dr. C. A. Jansen of the Cell and Immunology Group of Wageningen University (Wageningen, The Netherland), were maintained in Roswell Park Memorial Institute (**RPMI**) 1640 medium containing GlutaMAX, phenol red, and 25 mM HEPES (Gibco, Thermo Fisher Scientific, Waltham, USA) and supplemented with 8 % v/v heat inactivated foetal calf serum (Gibco) and 1 % v/v Penicillin-Streptomycin 100 U/ml (Gibco) both. HD11 cells were incubated at 41°C under 5 % CO_2_ in a humidified atmosphere. Cells were subcultured twice a week by preliminary washing with prewarmed (41°C) Dulbecco’s Phosphate Buffer Saline without calcium and magnesium (**DPBS**; Gibco), followed by two 5 min washes with DPBS supplemented with 0.5 mM EDTA. The cell suspension was centrifuged (200 × *g*; 5 min; room temperature) before reseeding the cells.

### Cell stimulation

*Escherichia coli* LPS stock solutions were prepared by dissolving *E. coli* LPS (serotype O55:B5, cat. no. L2880; Sigma Chemical Co. St. Louis, MO, USA) in RPMI 1640 at 1 mg/ ml and stored at −20°C until use. LPS stock solutions were thawed and diluted to the appropriate concentration immediately before use. Solutions containing LPS were prepared by dilution in RPMI 1640 containing GlutaMAX, phenol red and 25 mM HEPES (Gibco) and supplemented with 1 % v/v Penicillin-Streptomycin at 100 U/ml both (Gibco). For the experiments, cells at 80-90 % confluency were subcultured in 96-well-culture plates at 2 × 10^5^ cells in 200 µl per well (Cellstar, Greiner Bio-One, Kremsmünster, Austria). After overnight incubation, the medium was replaced by an equal volume of 0, 3, 30 and 300 ng/ml LPS solution for 5 and 20 h. The LPS doses and incubation time were determined based on a dataset of van Baal (unpublished) showing that in comparison to 1 ng/ml, 3 ng/ml LPS was required to observe a production on NO quantifiable at 6 and 16 h. The dose of 300 mg/ml corresponded to the dose were the plateau in NO production was reached without hampering the cell viability which was not maintained when the cells were treated with 1000 ng/ml LPS. Thus, the two concentrations represent the borders of the whole range where cell activation could be measured based on NO production and where viability is not hampered. The 5 h time point was chosen to reflect the early transcriptome reprogramming when NO start to be produced even at the lowest LPS dose. Additionally, in LPS-stimulated HD11 cells, the maximum or minimum expression of genes was between 4 and 6 h of stimulation (Ciraci et al., 2010; Slawinska et al., 2016a) giving the highest likelihood of detecting activated and repressed pathways in the early phases of the cell response.

### Measurement of nitric oxide production

The NO production by HD11 cells was determined indirectly, after 20 h of incubation, by measuring the production of the reactive nitrogen intermediate nitrite, with the Griess colorimetric assay (Green et al., 1982). Briefly, 50 μl cell supernatant was mixed with an equal volume of Griess reagent and incubated for 10 min at room temperature. Griess reagent was obtained by mixing a solution of 0.2 % w/v naphthylethylenediamine dihydrochloride in MilliQ water with a solution of 2 % w/v sulfanylamid in 5 % H_3_PO_4_. Absorbance was read at 540 nm in a Multiskan GO spectrophotometer (Thermo Fisher Scientific) and nitrite concentration was determined by means of a nitrite linear calibration curve.

### Measure of cell viability

HD11-cell viability was evaluated using Alamar blue, a non-toxic cell-permeable solution (Rampersad, 2012). Briefly, after 20 h incubation, the medium in the culture plate was emptied and immediately replaced by 100 µl of a diluted Alamar blue solution. Stock solution of Alamar blue was made by dissolving 1 g of Resazurin Sodium Salt (BioReagent, R7017, Sigma-Aldrich Chemie GmbH, Schnelldorf, Germany) in 100 mL sterile PBS (Gibco) followed by sterile filtering through a 0.22 µm pore size syringe filter (Acrodisc, Pall Laboratory Corporation, New York, NY, USA) and stored at −20°C protected from light until further use. For use, the stock solution was diluted 250 time in prewarmed culture medium without foetal calf serum. Cells with Alamar blue solution were incubated at 41°C in 5 % CO_2_ and 95 % humidity for 50 min in the dark. Reduction of Alamar blue was quantified by measuring the fluorescence at 590 nm following excitation at 560 nm (Multiscan GO, Thermo Fisher Scientific).

### Gene expression analysis

Gene expression analyses were performed after 5 h incubation with stimulation solutions. Cells were lysed with RA1 buffer and RNA was isolated using a RNA minikit (Thermo Fisher Scientific). RNA quantity and quality were checked by Nanodrop (ND-1000; Thermo Scientific). Subsequently, 500 ng total RNA was reverse-translated to complementary (c)DNA with Superscript III (Thermo Fisher Scientific) and T1 thermocycler (Biometra, Göttingen, Germany) for 5 min at 25°C followed by 60 min at 50°C, 15 min at 55°C, 15 min at 70°C and kept at 4°C until storage at −30°C. 1/50 diluted cDNA aliquots were quantified with QuantStudio 5 (Thermo Fisher Scientific) and SensiFast SYBR Lo-ROX kit (Bioline, Paris, France). Briefly, 1 μL of each primer (5 μM), 10 μL SensiFast mix and 4 μL water were added to 5 μL of cDNA sample. Primers’ sequences are presented in Table 1. The PCR protocol was: 2 min holding at 95°C followed by 35 to 40 cycles of 15 s at 95°C and 30 s at 60°C. The PCR was followed by melting curve analysis to confirm that the primers induced a specific amplification of its target mRNA. Relative expression was calculated using the 2^ΔΔCT^ method and GADPH, RLPO, PPIA as housekeeping genes.

**Table 1:** Primers used for RT-qPCR.

Target^1^	Forward 5′−3′	Reverse 5′−3′
GADPH	ATCCCTGAGCTGAATGGGAAG	AGCAGCCTTCACTACCCTCT
RPLP0	TTGGGCATCACCACAAAGATT	CCCACTTTGTCTCCGGTCTTAA
PPIA	CCCGTCGTGTTCTTCGACAT	CCCTTGTAGCCAAATCCCTTCT
IL-12b	CCCAGATGCTGGCAACTACA	GAACGTCTTGCTTGGCTCTTT
iNOS	CTACCAGGTGGATGCATGGAA	ATGACGCCAAGAGTACAGCC

1 GAPDH: Glyceraldehyde-3-phosphate dehydrogenase; RPLP0: Ribosomal protein lateral stalk subunit P0; PPIA: Peptidylprolyl Isomerase A; IL: Interleukin; NOS: inducible nitric oxide synthase.

### Transcriptome analyses

The transcriptome was analysed in a subset of samples with 4 replicates each. First, RNA quantity and quality were confirmed by using the Qubit (Thermo Fisher Scientific) and the Bioanalyzer (Agilent, Santa Clara, USA), respectively. Then, samples were sent to GENEWIZ/Azenta (Leipzig, Germany) for RNAseq. Briefly, PolyA selection was performed via NEBNext Poly(A) mRNA Magnetic Isolation Module (New England Biolabs, Ipswich, USA). Then, the NEBNext Ultra II RNA Library Preparation Kit (New England Biolabs) was used according to manufacturer protocol, and samples were sequenced with an NovaSeq 6000 (Illumina, San Diego, USA) with the 2 × 150 bp configuration and ∼30 M pair-ends reads per sample.

The preprocess of RNAseq data was performed with Galaxy (Afgan et al., 2018). First, data were trimmed with Sickle v1.22.2 with default parameters. Then, reads were aligned to the Gallus gallus bGalGal1.mat.broiler.GRCg7b reference genome and version 110 for known spliced sites (Ensembl) with HISAT2 v.2.2.1 using default parameters except for intron length (set to 10000). Aligned reads were counted with htseq-count v0.9.1 with union mode for overlapping reads and other parameters as default. Then, the low expressed genes were filtered with filterByExpr (edgeR v.3.42.4) (Robinson et al., 2010) using the default parameters and the genes without a non-ambiguous NCBI-Ensembl gene annotation were removed. Then, library sizes were normalised per treatment group and the negative binomial generalized model was built using the normLibSize and glmQLFit functions (edgeR), respectively.

### Statistical analyses

The effects of LPS on nitric oxide production, cell viability and RT-qPCR measured gene expression were analysed by ANOVA, with LPS as a factor effect and a random replicate effect, followed by a Tukey posthoc test. Residuals normality was controlled by Shapiro test. Data analyses were performed with R v.4.3.1. software (R Core Team, 2023). The effects of LPS on the transcriptome were analysed by differential expression analysis with edgeR v.3.42.4 (Robinson et al., 2010). Comparisons between treatments for foldchange and p-values were performed with glmQLFTest (edgeR) and genes with a false discovery rate < 0.05 were considered differentially expressed. A multidimensional scaling (**MDS**) was performed with the plotMDS function from package limma, v. 3.56.2 (Ritchie et al., 2015) and all genes. KEGG pathways gene set enrichment analysis between treatments was performed on the complete gene list ordered by foldchange with gseKEGG from package clusterProviler v.4.8.2. (Wu et al., 2021) using the *Gallus gallus* reference, a p-value cut-off of 0.05 and a seed. The immune gene and transcription factors (**TFs**) regulatory networks analysis using the exp2grn function of BioNERO R package v.1.8.7 (Almeida-Silva and Venancio, 2022) using 5000 trees, 10 splits, and a seed. The differences were considered significant at p-values or adjusted p-values less than 0.05.

## Results

**Fig. 1:**
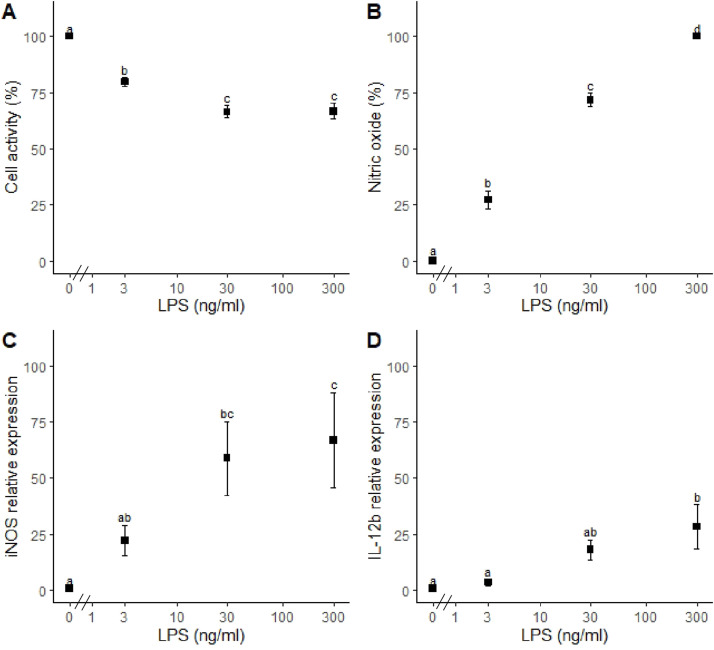
Effect of escherichia coli lipopolysaccharide (LPS) on avian HD11 macrophage-like cells viability, nitric oxide (NO) production, and inducible nitric oxide synthase (iNOS) and interleukin 12b (IL-12b) gene expression. HD11 cells were treated with medium supplemented with LPS (0, 3, 30 and 300 ng/ml) for 20 h and NO in the supernatant was quantified prior to the measurement for cell viability by Alamar Blue assay after 1 h incubation. Gene expression was measured at 5 h incubation. Data are represented as means ± standard error of the mean (SEM) of 5 independent experiments.

The doses of 3, 30 and 300 ng/ml LPS yielding a low (∼25 % of the maximal NO production), middle (∼75 % of the maximal NO production) and high effective dosages (100 % of the maximal NO production), respectively (Fig. 1B) were used. The NO production by LPS-stimulated HD11 cells at 20 h was shown to be dose-dependent (Fig. 1B) with a maximum of 15.0 ± 3.6 µM NO (mean ± SEM; 4 wells) at 300 ng/ml LPS. Cell viability was decreased dose-dependently by LPS from 0 to 30 ng/ml (−20 and −38 % at 3 and 30 ng/ml compared to the negative control), but no further reduction of cell viability was observed at 300 compared to 30 ng/ml LPS (Fig. 1A). In parallel, the mRNA expression of iNOS, which encodes the NO producing enzyme, reached a plateau at 30 ng/ml LPS (Fig. 1C). The mRNA expression of the interleukin 12b (**IL-12b**) was measured as an additional marker of the macrophage pro-inflammatory response and only the expression level at 300 ng/ml LPS was different, compared to 0, 3 and 30 ng/ml LPS (Fig. 1D). Based on these measures the LPS doses of 3 and 300 ng/ml were regarded to be low and high effective doses and were, therefore, used further to study transcriptomic reprogramming of avian macrophages at low and high LPS doses.

The total number of reads per sample ranged from 34 to 81 million. The sequenced reads were then mapped to the reference chicken genome with an overall alignment rate of 72.4 to 76.3 %. In total 30108 genes were mapped, out of which 10796 remained after filtering of the very low-expressed genes (below 10 copies). The remaining genes were used for further analysis as presented hereafter.

**Fig. 2:**
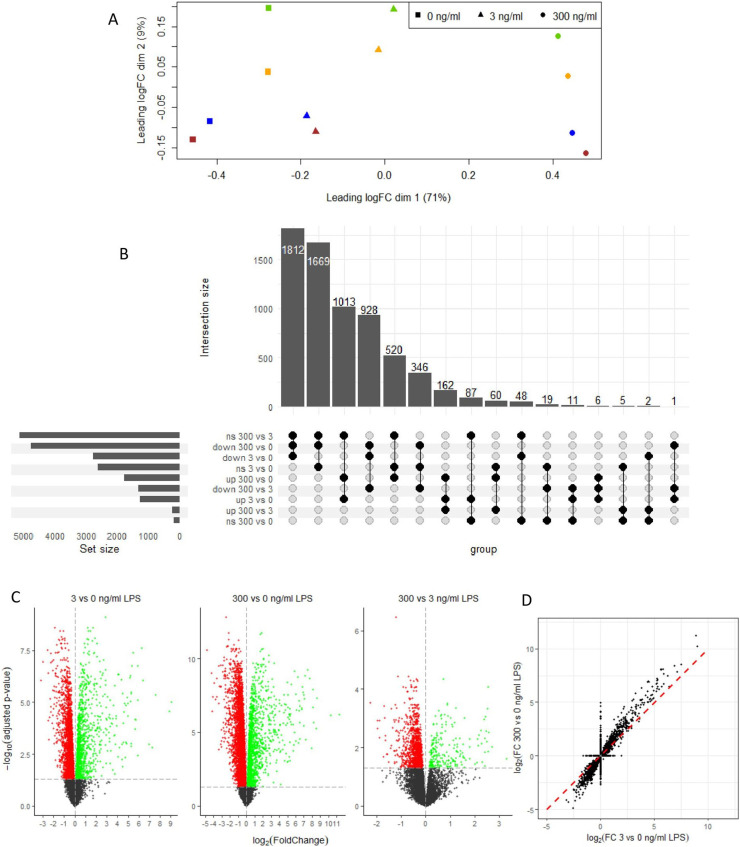
Effect of *Escherichia coli* lipopolysaccharide (LPS) on avian HD11 macrophage-like cells gene expression. (A) MDS-plot with one colour per replicate. (B) Upset plot showing among the differentially expressed genes, at 3 vs 0, 300 vs 0 and 300 vs 3 ng/ml LPS, the number of shared and different upregulated (up), downregulated (down), and not significantly different (ns) genes. (C) Volcano plot of the differential mRNA expression for the indicated comparisons. Significant threshold was set at discovery rate (FDR) > 0.05 (horizontal dashed line). (D) correlation between log2(Fold change) for the comparisons 3 vs 0 and 300 vs 0 ng LPS/ml. Comparisons with a FDR > 0.05 were set to 0.

A multidimensional scaling (**MDS**) was performed (Fig. 2A) and revealed a good clustering of the treatment groups toward the x-axis which explained a high proportion of the variability (71 %) while the replicates segregated toward the y-axis and explained a minor part of the variability (9 %). The differentially expressed gene (**DEG**) analysis was conducted between all treatments. Compared to the negative control, the 3 and 300 ng LPS /ml induced upregulation of 11.8 and 16.3 % of the genes, and 25.8 and 44.1 % of the genes were downregulated (Fig. 2B and C). Interestingly, this differential gene expression profile showed that LPS caused a 2-3 fold higher number of downregulated than upregulated genes. A comparison of the fold-change of the genes affected at 3 and 300 ng LPS/ml showed that LPS at 300 ng/ml was exacerbating the effect of 3 ng/ml (Fig. 2D). Among the 1181 genes upregulated by 3 and 300 ng LPS/ml when compared to the negative control, 1004 genes (85.0 %) were more upregulated in the 300 ng/ml condition out of which 162 genes (13.7 %) showed a significant upregulation for 3 vs 300 ng LPS/ml (Fig. 2B and C). Similarly, among the 2740 genes downregulated by 3 and 300 ng/ml LPS when compared to the negative control, 2623 genes (95.8 %) were more downregulated in the 300 ng/ml condition and 928 genes (33.9 %) showed a significant difference for 3 vs 300 ng/ml LPS (Fig. 2B).

**Fig. 3:**
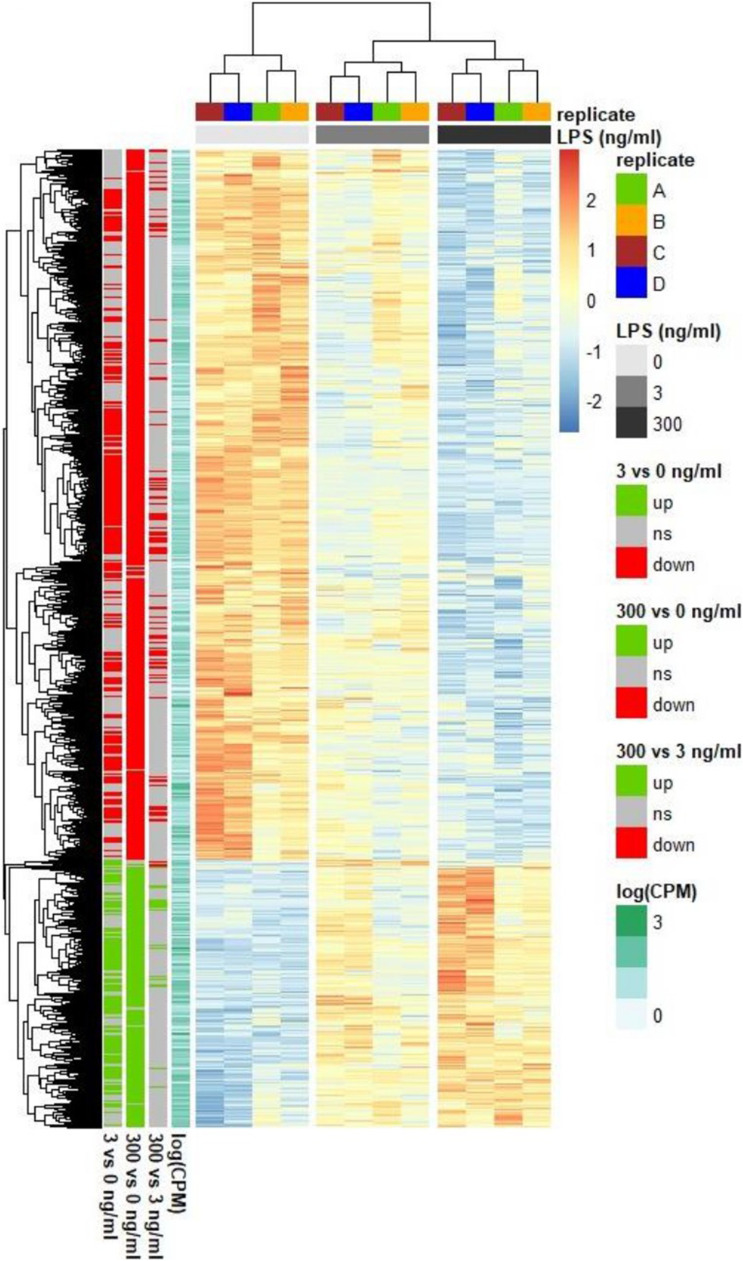
Heatmap of the mRNA gene expression profile from chicken HD11 cells treated with 0, 3 and 300 ng/ml *Escherichia coli* lipopolysaccharide (LPS). Expression is normalised within each row. Only the 6477 genes with a false discovery rate < 0.05 for at least one comparison (3 vs 0, 300 vs 0 or 300 vs 3) are represented. ns stands for non-significant difference, up for upregulation and down for downregulation. CPM: count per million.

**Fig. 4:**
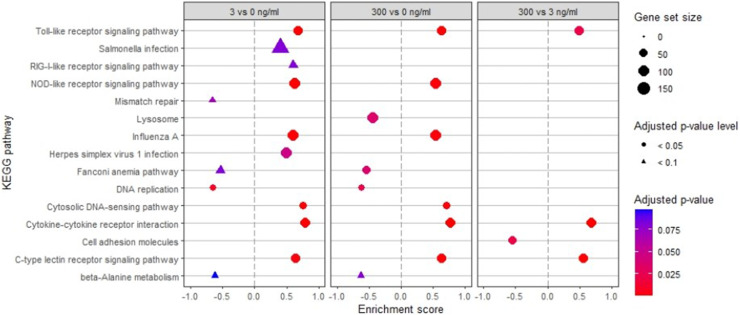
Gene set enrichment analysis of KEGG pathways. Only pathways with an adjusted p-value < 0.1 are presented.

Genes that were differentially expressed (**DEG**) in at least one of the three conditions were plotted in a heatmap (Fig. 3), together with a hierarchical clustering dendrogram on gene expression level and samples, showing which samples had a comparable gene expression pattern. This approach showed a clear treatment effect on, up and down regulated genes. To further elucidate the broad functions of the DEG, a gene set enrichment analysis of KEGG pathways was performed with all pathways having an adjusted p-value < 0.1 for the enrichment score (Fig. 4). Compared to the negative control, LPS at 3 and 300 ng/ml, up- and down-regulated similar pathways. However, three pathways were more affected at 300 than at 3 ng/ml LPS and all were enriched: the toll-like receptor signalling pathway, the cytokine-cytokine receptor interaction and the C-type lectin receptor signalling pathway. The maximal effect for the NOD-like receptor signalling pathway, the cytosolic DNA-sensing pathway (both enriched), and DNA replication (de-enriched) was already achieved at 3 ng/ml LPS, with no further amplification observed at 300 ng/ml LPS. At 300 vs 3 ng/ml LPS, the cell adhesion molecule pathway was the only one with a significant negative enrichment score.

**Fig. 5:**
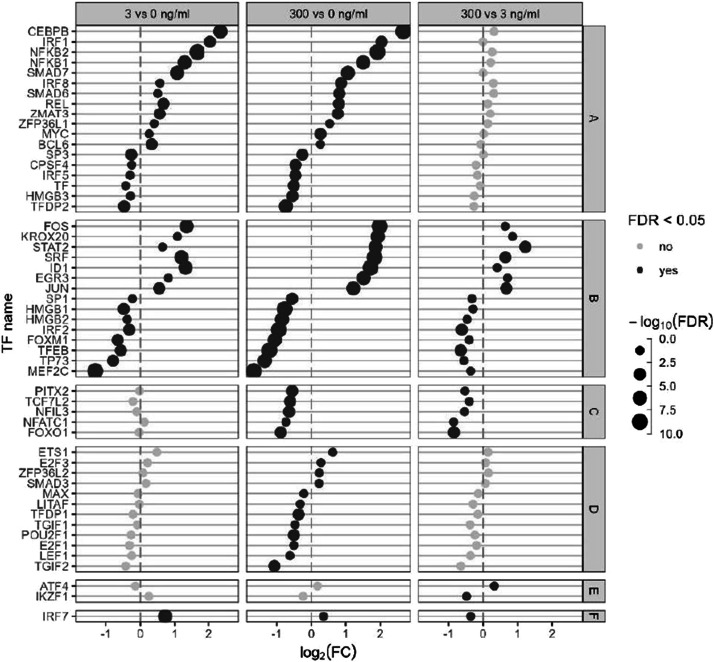
Effect of a low and a high dose of *Escherichia coli* lipopolysaccharide (LPS) on avian HD11 macrophage-like cells mRNA expression of immune related transcription factors (TFs). Represented TFs have a false discovery rate < 0.05 in at least one comparison. TFs were separated in six categories: A: maximal significant changes reached at 3 ng/ml LPS; B: dose-dependent increase up to 300 ng/ml LPS; C: significant effect of 300 ng/ml LPS compared to the 0 and 3 ng/ml LPS; D: significant effect of 300 ng/ml LPS compared to the 0 but not 3 ng/ml LPS; E: significant effect at 3 ng/ml LPS only; F: higher fold change at 3 vs 0 compared to 300 vs 0 ng/ml LPS.

In order to examine the response of the HD11 cells related to the immune processes, we identified all genes that were associated with GO:0002376 “immune system process” as well as the one related to KEGG immune system subcategory 04620, 04621, 04622, 04623, 04625 and 04672, KEGG membrane transport subcategory 02010, KEGG signal transduction subcategory 04010, 04020, 04060, 04350 and 04370, KEGG infectious disease subcategory 05132, 05164 and 05168, KEGG cellular processes subcategory 04115, 04218, 04110, 04142, 04145 and 04146. Of these genes, the TFs and co-TFs, as identified with the AnimalTFDB v4.0 software (Shen et al., 2022), were separated from the other genes. To further elucidate the expression pattern of the TFs, the one with a false discovery rate (**FDR**) < 0.05 for at least one comparison were separated in six categories (A to F) and represented in Fig. 5. Twelve TFs reached a maximal expression at 3 ng LPS/ml while 6 reached their minimal expression as shown by a FDR < 0.05 for 3 vs 0 and 300 vs 0 but not for 300 vs 3 (A). Seven TFs were up-regulated dose-dependently from 0 to 300 ng LPS/ml, while eight were down-regulated dose-dependently as shown by a FDR < 0.05 for all comparisons (B). Five TFs were DE by 300 but not 3 ng LPS/ml and were all down regulated (C). Twelve TFs were affected only at 300 vs 0 ng LPS/ml (D) and two TFs were affected only at 300 vs 3 ng LPS/ml (E) with low fold change and represent minor effects. Interestingly, IRF7 expression was the only TF increased significantly more by 3 than by 300 ng/ml LPS (F).

**Fig. 6:**
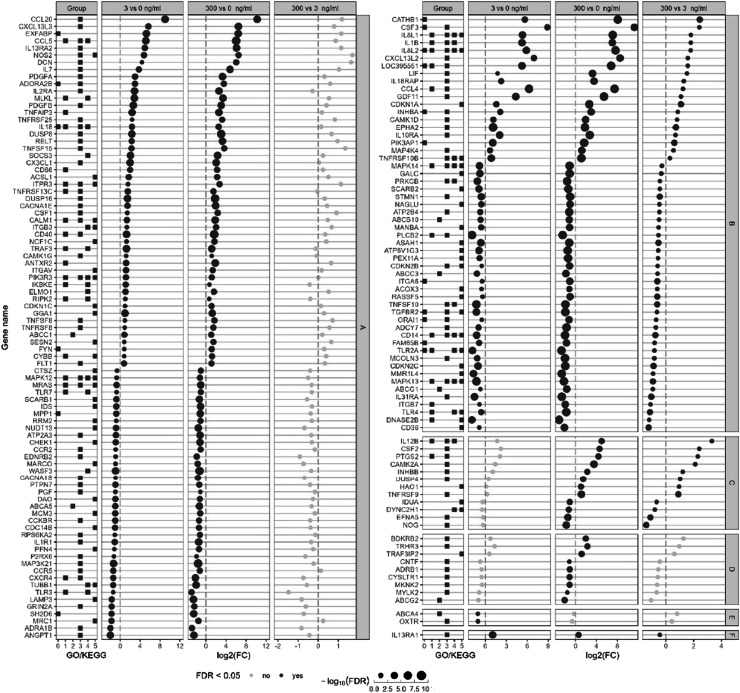
Effect of a low and a high dose of *Escherichia coli* lipopolysaccharide (LPS) on avian HD11 macrophage-like cells relative mRNA expression of immune related genes excluding transcription factors and transcription co-factors were excluded. Represented genes have false discovery rate < 0.05 and a |log2(fold change)| > 1 in at least one comparison. Gene were separated in six categories: A: maximal significant changes reached at 3 ng/ml LPS; B: dose-dependent increase up to 300 ng/ml LPS; C: significant effect of 300 ng/ml LPS compared to the 0 and 3 ng/ml LPS; D: significant effect of 300 ng/ml LPS compared to the 0 but not 3 ng/ml LPS; E: significant effect at 3 ng/ml LPS only; F: higher fold change at 3 vs 0 compared to 300 vs 0 ng/ml LPS. Group GO/KEGG: 0: GO:0002376 immune system process; 1: KEGG immune system 04620, 04621, 04622, 04623, 04625 and 04672; 2: KEGG membrane transport 02010; 3: KEGG signal transduction 04010, 04020, 04060, 04350 and 04370; 4: KEGG infectious disease 05132, 05164 and 05168; 5: KEGG cellular processes 04115, 04218, 04110, 04142, 04145 and 04146.

**Fig. 7:**
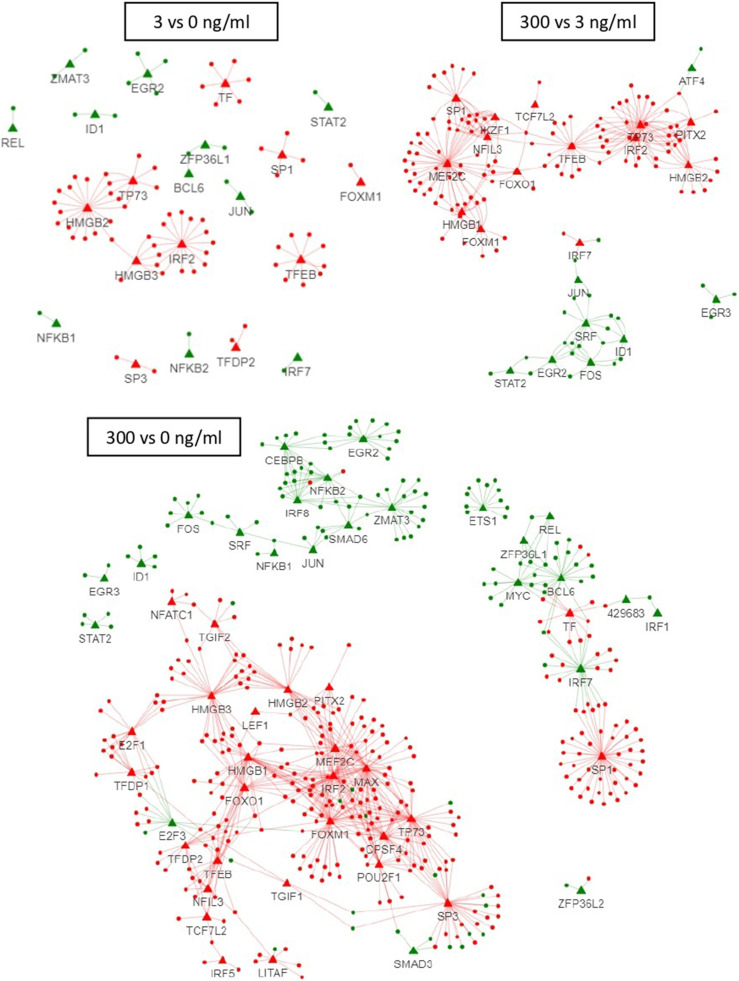
Immune gene and transcription factors (TFs) regulatory network interaction pattern at low and high dose of *Escherichia coli* lipopolysaccharide (LPS) in avian HD11 macrophage-like cells. Per comparison, the genes (circles) and TFs (triangles) with a false discovery rate > 0.05 were included in the regulatory network inference. Red: downregulated genes and TFs. Green: Upregulated genes and TFs.

For other immune-related genes, only the one with a FDR < 0.05 and a |log_2_(fold change)| > 1 in at least one comparison were included in the analysis and represented in Fig. 6. The genes were clustered in six categories: maximum up- or down-regulation in expression reached at 3 ng LPS/ml already (A: 50 up and 38 down), effect of 3 ng LPS/ml exacerbated by 300 (B: 19 up and 35 down), expression affected at 300 ng/ml only (C: 8 up and 4 down), DE only at 300 vs 0 ng LPS/ml (D: 3 up and 6 down), DE only at 3 vs 0 ng LPS/ml (E: 2 down) and DE oppositely between 3 and 300 ng LPS/ml (E: 1 down). Finaly, we built regulatory networks between the differentially expressed immune-related genes and TFs per treatment to identify TFs playing a possible role in the HD11 mediated immune-response (Fig. 7). Most connected TFs at the 3 vs 0 ng/ml LPS comparison were HMGB2, HMGB3, IRF2, TF and TP73, and were all downregulated and also present in the 300 vs 0 comparison. With the 300 ng/ml LPS condition, ten extra TFs appeared having many connections, which were PITX2, TCF7L2, NFIL3, FOXO1, HMGB1, MEF2C, IKZF1, PITXA, SRF and FOS.

## Discussion

As in mammals, macrophages are key cells of the chicken immune system, which contribute significantly to protection against infection. For this reason, macrophages are intensively studied to understand the responses to immune stimulating molecules such as LPS. However, thus far, the transcriptomic reprogramming of chicken macrophages *in vitro* has been examined only with a single dose of 100 ng/ml LPS (Zhang et al., 2021). In murine macrophages, low and high LPS inflammatory stimuli activated different cellular pathways (Naler et al., 2022). To investigate whether low and high dose of LPS also induce differential transcriptomic activation in chicken macrophages, we exposed the chicken macrophage-like HD11 cell line to different doses of LPS as a prerequisite for further experiments. Based on NO production, iNOS and IL-12b mRNA expression, we selected a low (3 ng/ml) and high dose (300 ng/ml) of LPS for the subsequent transcriptomic analysis. The low dose corresponds to the lowest dose showing 25 % of the maximal NO production while the high dose corresponds to the plateau phase (*d.i.* 100 % of the maximal NO production) of the response. Following this approach, the present study nicely extends the work of Zhang et al. (2021). Both studies analysed reprogramming of the transcriptome at comparable time points post LPS stimulation (5 vs 6 h), which examines the early inflammatory response.

Coherently, both LPS doses increased the expression of the following genes characterised by the proinflammatory M1 phenotype of macrophages: namely chemokine ligand 20 (**CCL20**), CD40, CD86, iNOS, IL-1b, IL-7, the two IL-8 subunits L1 and L2, and IL18. The CCL20 was by far the most upregulated gene among the one reaching their maximal expression at 3 ng/ml LPS. The chemokine CCL20 is multifunctional acting as a chemoattractant for immune cells, participating in inflammatory diseases and helping to maintain tissue homeostasis (Li et al., 2022). The surface markers CD40 and CD86 are fundamental for activation of B and T cells (Koskela et al., 1998; Kothlow et al., 2008; Elgueta et al., 2009). The most dose-dependently upregulated genes were cathelicidin CATHB1 and colony-stimulating factor 3 (**CSF3**). The innate factor CATHB1 is one of the four avian β-defensins, which has antibacterial activity and an immune regulatory function (Cheng et al., 2015). Colony-stimulating factor CSF3, previously called myelomonocytic growth factor (**MGF**) (Gibson et al., 2009), is induced by LPS and may sustain iNOS expression (Laurent et al., 2001). Surprisingly, the latter two genes, despite being the most upregulated by LPS in our study, appear not to be reported as a LPS-responsive gene in chicken macrophages in the literature. Consistently with the upregulation of M1 inflammatory factors, immune-related KEGG pathways were significantly enriched: namely Toll-like, NOD-like, and C-type lectin receptor signalling, influenza A, cytosolic DNA-sensing and the cytokine-cytokine receptor interaction pathways. Many of these pathways have numerous genes in common. Only three KEGG pathways were further enriched by the high dose of LPS compared to the low dose, including Toll-like and C-type lectin receptor signalling pathways as well as the cytokine-cytokine receptor interaction. These findings suggest that a dose of 3 ng/ml LPS was already sufficient to evoke a pro-inflammatory phenotype in HD11-macrophages. This dose appears to be much lower than the relatively high doses used in many other studies (Gou et al., 2015; Slawinska et al., 2016b; Xu et al., 2022). The higher LPS dose required in other studies is likely due to the use of foetal calf serum in the culture medium during LPS stimulation, which reduces cell responsiveness (Kadowaki et al., 2013).

The transcriptomic analysis identified a set of immune-related TF reaching their maximal up- or down-regulation already at 3 ng/ml. The TFs with highest fold change was CCAAT enhancer binding protein beta (**CEBPB**) followed by interferon regulatory factor 1 (**IRF1**). Surprisingly, those two TFs were not picked up as key regulators in the 3 vs 0, but only in the 300 vs 0 regulatory network, potentially because their downstream genes were not enough differentially expressed. In HD11 cells, IRF1 was reported to promote the macrophage response to viruses (Li et al., 2015). In mammals, CEBPB mRNA encodes for the C/EBPβ protein, which promotes inflammation in LPS stimulated macrophages (Ren et al., 2023). However, the role of C/EBPβ in mammalian inflammation is controversial (Ren et al., 2023), as studies have shown that CEBPB activation can also promote an anti-inflammatory M2 phenotype in macrophages (Ruffell et al., 2009; Lamkin et al., 2019). Despite using a different LPS source, accordingly to our results, Zimmermann et al. (2015) reported that *Salmonella Typhimurium* LPS increased C/EBPβ activity in HD11 macrophages. In absence of studies on identical *E. coli* LPS stimulation, this support our findings by indicating that LPS can increase CEBPB expression/ C/EBPβ activity in HD11 macrophages. It should be noted however that LPS from different bacterial species have been reported to elicit different activation responses in murine macrophages (Biedroń et al., 2016). On the network, CEBPB is connected to 14 upregulated genes (MST1R, CD82, SQSTM1, GGA1, SESN2, CD86, ANTXR2, HSP90AA1, ATP2A2, MDM4, LFNG, CSF1, PDGFA and TNFSF8), which may contribute to the inflammatory response of chicken macrophages. Accordingly, inter alia, CD82 promotes CD8+ *T* cell function (Kim et al., 2021), CD86 is involved in immune response to infection and regulate T cells activation (Sun et al., 2024), while MST1R is a macrophage receptor for macrophage-stimulating protein suppressing cell-mediated immune responses (Morrison et al., 2004), but most of these factors/ molecules remained to be investigated in the chicken. Species specific study are important because avian and mammalian intracellular pathways activated by LPS differ (Keestra and van Putten, 2008). In chicken TLR4 stimulation does not activate the MyD88-independent pathway (Keestra and van Putten, 2008), most likely because TRAM is lacking (Brownlie and Allan, 2011). Consequently some genes and TF that fulfill specific functions in mammalian cells may lack part of it in avians, and *vice versa*. Chicken TLR4 and its coreceptor, the associated myeloid differentiation protein-2 (**MD-2**), both required for LPS detection show polymorphisms in different chicken breeds (Ramesh et al., 2022). Such polymorphisms in HD11 cells may also influence their response to LPS. Indeed, chicken TLR4 polymorphisms have been associated with varia susceptibility to *Salmonella* infection (Leveque et al., 2003).

Five TF were downregulated only at the high LPS dose (PITX2, TCF7L2, NFIL3, NFATC1 and FOXO1). In mammalian macrophages, TCF7L2, also known as transcription factor 7-like 2 or TCF4, was associated with the polarization towards a immunosuppressive M2 phenotype (Li et al., 2021). Since LPS is known to induce polarization towards the M1 phenotype, it seems coherent that this gene was downregulated by LPS stimulation. The presence and some functions of TCF7L2, FOXO1 (forkhead box protein O1) and NFIL3 (nuclear factor, interleukin 3 regulated) gene product were confirmed *in vivo* in chicken (Keniry et al., 2014; Liu et al., 2021; Chi et al., 2021), but to our knowledge, their suggested involvement in the macrophage response to LPS was never reported. NFIL3 and NFATC1 (nuclear factor of activated T cells 1) are other TF with known immunosuppressive function in mammals (Kobayashi et al., 2011; Fric et al., 2012; Keniry et al., 2014), but such a role in chickens remains to be investigated. FOXO1 was reported to promote the M1 phenotype of mammalian macrophages (Fan et al., 2010; Yan et al., 2020; Rong et al., 2022), but was also reported to promote M2 polarization in mammalian macrophages (Rong et al., 2022) via CCL20 and CSF1 (colony stimulating factor 1) (Wang et al., 2020). FOXO1 was also reported to up-regulate TLR4 transcription and its proinflammatory pathway (Fan et al., 2010). Interestingly, PITX2 (paired like homeodomain 2), TCF7L2, NFIL3 (interleukin-3 binding protein 1), and FOXO1 are retrieved in the immune gene-TF interaction network for 300 vs 3, which tended to confirm their role in macrophage response to high dose of LPS. On the network, the downregulated TF, SP1 and SP3 are particularly connected to downregulated genes. Those TFs play a role in regulating the expression of anti-inflammatory cytokine IL-10 (Tone et al., 2000). The reduced expression of several M2 related TFs at the high dose of LPS only, suggests that the cell is highly dedicated to the pro-inflammatory functions. The lower upregulation of interferon factor 7 (**IRF7**) at the high LPS dose compared to the low dose, suggest the control of the M2-to-M1 polarity switch, as reported before in mammalian species (Cohen et al., 2014). We suggest that cells treated with the high LPS dose are more polarized into a M1 phenotype than cells treated with low LPS dose, and therefore less responsive to exogenous M2-inducing compounds. Conversely, macrophages treated with a low dose of LPS may remain more sensitive to stimuli that promote an immune-regulatory M2 phenotype. Such potential LPS dose-dependent differences in HD11 activation could be important when evaluating for the effects of exogenous dietary or pharmaceutical compounds, suggesting that the latter should be ideally assessed in interaction with low and high LPS doses.

In addition to the transcription factors PITX2, TCF7L2, NFIL3, and FOXO1, six others were also only retrieved in the 300 vs 3 ng/ml LPS network, namely HMGB1, MEF2C, IKZF1, and PITXA, which were downregulated, and serum response factor (**SRF**) and leucine zipper protein FOS, which were upregulated. In mammals, MEF2C (myocyte enhancer factor 2C) was identified as an enhancer of M1 macrophage polarization (Zhao et al., 2022), but its role may be limited (Cilenti et al., 2021). Downregulation of MEF2C in HD11 macrophages by LPS was also found by Zhang et al. (2021). In line with this, FOS, also known as c-FOS, was reported to suppress expression of pro-inflammatory cytokines and iNOS, in murine macrophages (Ray et al., 2006). Furthermore, FOS expression is notably positively regulated by the SRF (Chai and Tarnawski, 2002). Our observed higher expression of FOS and SRF may reflect a regulatory mechanism to avoid an overreaction of the cells. These data revealed new potential key TFs controlling the LPS response in chicken macrophages which will require further confirmation by dedicated studies.

Surprisingly, although the HD11 cells were stimulated by LPS for 5 h, we observed that compared to the number of up-regulated genes, a far greater proportion was down-regulated. By increasing the LPS dose from 3 to 300 ng/ml, an additional 72 % were significantly downregulated compared to the 3 vs 0 ng/ml (2790 genes). This downregulation is likely not an artefact due to the normalisation step in the data analysis. The downregulation of many genes partly opposed the results of Zhang et al. (2021), who reported a greater proportion of upregulated (66 %) genes compared to downregulated ones (44 %). Furthermore, they found overall less DEG (1378) for a comparable total number of genes expressed (15883) in HD11 cells stimulated with a single LPS dose of 100 ng/ml. However, those authors used 15 % serum supplement, which is known to strongly affect the cell response to exogenous stimuli (Kadowaki et al., 2013). Based on our results, it seems that to sustain the stimulation of proinflammatory pathways, especially at high LPS concentrations, the cells needed to downregulate many functions. Among the downregulated genes, we found the toll-like receptors TLR2A, TLR3, TLR4 and TLR7. Downregulation of these pattern recognition receptors may prevent the cells for an overactive pro-inflammatory response towards exogenous components (Nahid et al., 2011). Unfortunately, despite testing with multiple pathways enrichment techniques, we could not pick up the role of the downregulated genes. A partial explanation of this is the underrepresentation of those genes in the pathways database (GO, KEGG) compared to the one of the inflammatory responses. The KEGG beta alanine metabolism, DNA replication, and Fanconi anaemia pathway were downregulated for both 3 vs 0 and 300 vs 0 but not for 300 vs 3 ng/ml LPS and represented a small set of genes compared to the high number of downregulated genes and did not pick up the additional effects of the 300 compared to the 3 ng/ml. Also, the databases for enrichment analysis are not optimal for the chicken (e.g. KEGG misses the NF-kB pathway for chicken). Also, TF enrichment analysis would have been helpful to understand our results better, but there are no tools available for chicken. This massive downregulation of genes in stimulated macrophages remains poorly investigated. Dedicated studies should elucidate whether this downregulation is necessary to express and maintain their pro-inflammatory phenotypes in *in vitro* experiments. It would be important to confirm whether this extensive downregulation of genes also occurs in chicken macrophages within 5 h after LPS challenge *in vivo.* Although immortalized macrophage-like cell lines provide a convenient screening tool (Paradowska et al., 2022), they do not fully represent primary macrophages, the tissue environment in which they function, or the systemic context of an in vivo immune response. The findings of the current study should therefore be followed up using primary macrophages and *in vivo* experiments.

## Conclusions

We compared the transcriptome of chicken macrophage-like cells in response to a low and high dose of LPS. After 5 h stimulation, it appeared that a greater proportion of genes was downregulated than upregulated. Our study offers the first detailed mapping of the HD11 macrophage-like response to both low and high doses of LPS. It is also a good start for further research, especially to validate the roles of many genes and transcription factors that have only been studied in mammals so far. Our results indicate that while the expression patterns of transcription factors and immune-related genes are largely consistent between low and high LPS doses, the response is significantly more intense at the highest dose. This heightened response may affect the sensitivity of the cells to dietary compounds. It is therefore recommended to use multiple LPS doses in *in vitro* studies examining the effects of dietary or pharmaceutical compounds on inflammation in chicken macrophages.

## Funding

This study was funded by Idena (Sautron, France), and the “Association Nationale Recherche Technologie” (ANRT) through the Convention Industrielle de Formation par la RecherchE (CIFRE) grant no. 2021-0384. The funding bodies played no role in the design, analysis and reporting of the study.

## Availability of data and materials

The datasets used and analysed during the current study are available from the corresponding author on reasonable request. Sequence data that support the findings of this study have been deposited in the NCBI Sequence Read Archive (SRA) database under the accession number PRJNA1153035.

## CRediT authorship contribution statement

**Samuel C.G. Jansseune:** Writing – review & editing, Writing – original draft, Visualization, Project administration, Methodology, Investigation, Funding acquisition, Formal analysis, Data curation, Conceptualization. **Jürgen van Baal:** Writing – review & editing, Supervision, Project administration, Methodology, Conceptualization. **Fany Blanc:** Writing – review & editing, Supervision, Investigation. **Aart Lammers:** Writing – review & editing, Supervision, Project administration, Conceptualization.

## Disclosures

Although one of the authors (SCGJ) was a PhD candidate employed by Idena, the authors attest that they were completely free to independently design the study and collect, analyse and interpretate the data as well as write the manuscript.
